# Supplementary material for: Dielectric Properties Investigation of Metal–Insulator–Metal (MIM) Capacitors
Source: Molecules. 2022 Jun 20;27(12):3951. doi: 10.3390/molecules27123951 (PMC9227371; doi:10.3390/molecules27123951)
Supplement: Supplementary file 1 [file molecules-27-03951-s001.zip › molecules-1732518-supplementary.pdf]

molecules

Supporting Information

Dielectric properties investigation of metal-insulator-metal (MIM) capacitors

Li Xiong[1], Jin Hu[1], Zhao Yang[2,3,*], Hang Zhang[4] and Guanhua Zhang[1,*]

[1]State Key Laboratory of Advanced Design and Manufacturing for Vehicle Body, College of Mechanical and Vehicle Engineering, Hunan University, Changsha 410082, China
[2]Guangdong Fenghua Advanced Technology Holding Co., Ltd., Zhaoqing, 526060, China
[3]State Key Laboratory of Advanced Material and Electronic Components, Zhaoqing, 526060, China
[4]Key Laboratory of Applied Surface and Colloid Chemistry, Ministry of Education, School of Chemistry and Chemical Engineering, Shaanxi Normal University, Xi'an 710119, P. R. China
* Correspondence: authors: Yangzhao@china-fenghua.com (Z. Yang); guanhuazhang@hnu.edu.cn (G. Zhang)

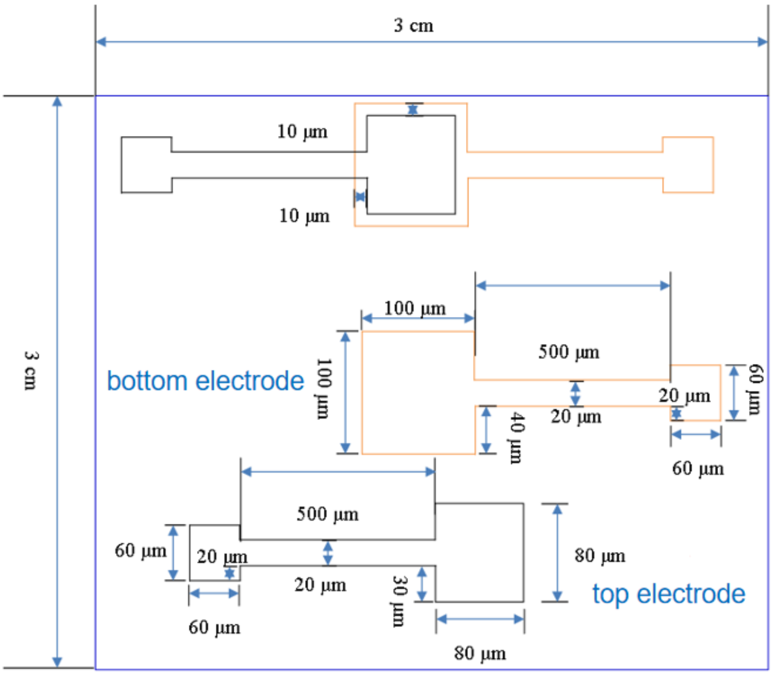

Figure S1. MIM capacitor electrode structure pattern design, arrange 30 unit patterns in a 3 cm × 3 cm area to form an array.

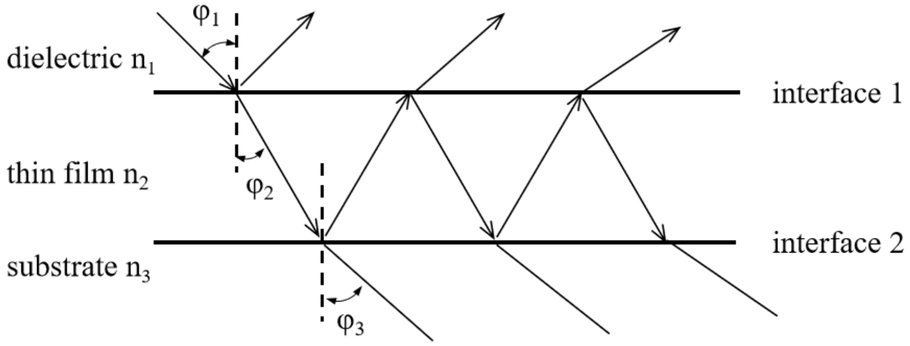

Figure S2. Reflection and refraction of light on thin film and substrate systems.

When a beam of light hits the film surface, multiple reflections and refractions are formed on the interface 1 and interface 2, and each reflected light and refracted light produce multi-beam interference respectively, whose interference results reflect the optical properties of the film. According to the law of refraction and reflection of light, combined with the Fresnel formula, the following relationship can be obtained as follows:

$$tg\varphi\ e^{i\Delta}=f(n_2,\ \varphi_1,\ n_3,\ d,\ \lambda) \quad \text{(S1)}$$

where φ_1 is the incident angle, n_3 is the refractive index of the substrate, and λ is the wavelength of the incident light, all of which are constants. φ and Δ can be obtained through experimental tests. Ellipsometer is an optical testing technique used to analyze the reflection (or transmission) of light from the surface of a sample. Ellipsometer measures two important parameters (φ, Δ), which represent the amplitude ratio φ and phase difference Δ between p-polarized light and s-polarized light, respectively. The thickness of the test film is obtained by the law of refraction of light and the Fresnel formula. Call the Cauchy model in the ellipsometry spectrometer to compare with the experimental test data.

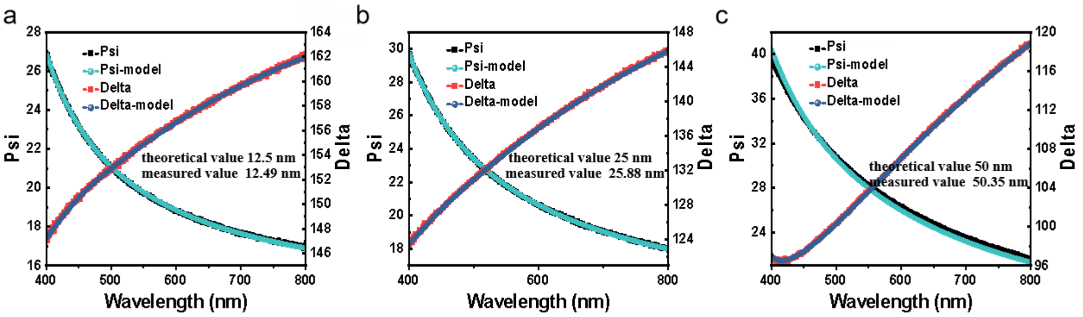

Figure S3. The dielectric capacitor thicknesses of 12.5 nm, 25 nm and 50 nm Al_2O_3 are completed by ALD and measured with a spectroscopic ellipsometer. (a) 12.5 nm Al_2O_3 dielectric film thickness test result. (b) 25 nm Al_2O_3 dielectric film thickness test result. (c) 50 nm Al_2O_3 dielectric film thickness test result.

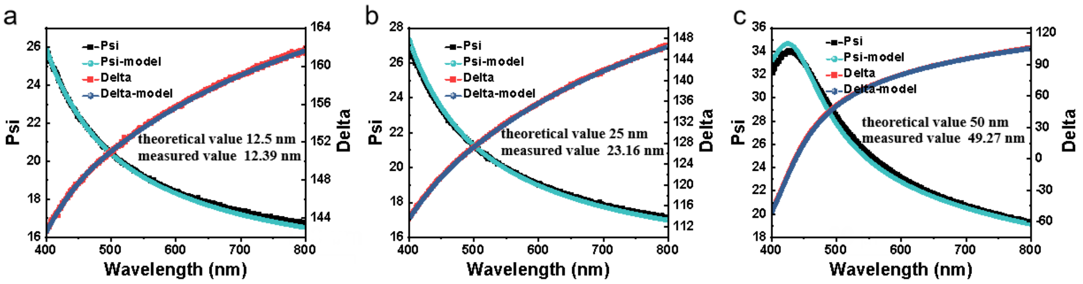

Figure S4. The dielectric capacitor thicknesses of 12.5 nm, 25 nm and 50 nm TiO_2 are completed by ALD and measured with a spectroscopic ellipsometer. (a) 12.5 nm TiO_2 dielectric film thickness test result. (b) 25 nm TiO_2 dielectric film thickness test result. (c) 50 nm TiO_2 dielectric film thickness test result.

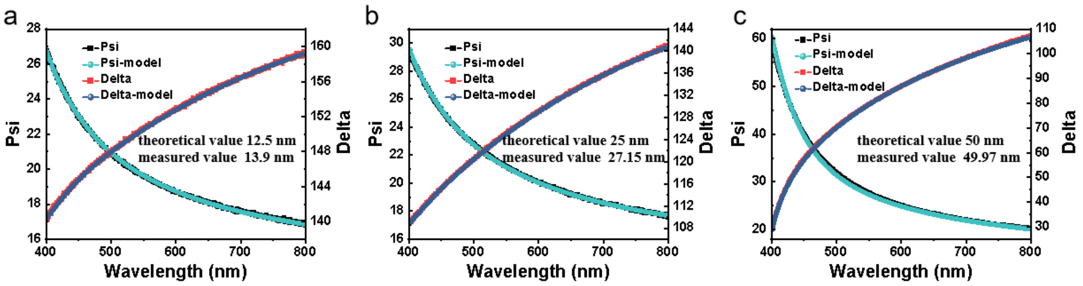

Figure S5. The dielectric capacitor thicknesses of 12.5 nm, 25 nm and 50 nm HfO_2 are completed by ALD and measured with a spectroscopic ellipsometer. (a) 12.5 nm HfO_2 dielectric film thickness test result. (b) 25 nm HfO_2 dielectric film thickness test result. (c) 50 nm HfO_2 dielectric film thickness test result.

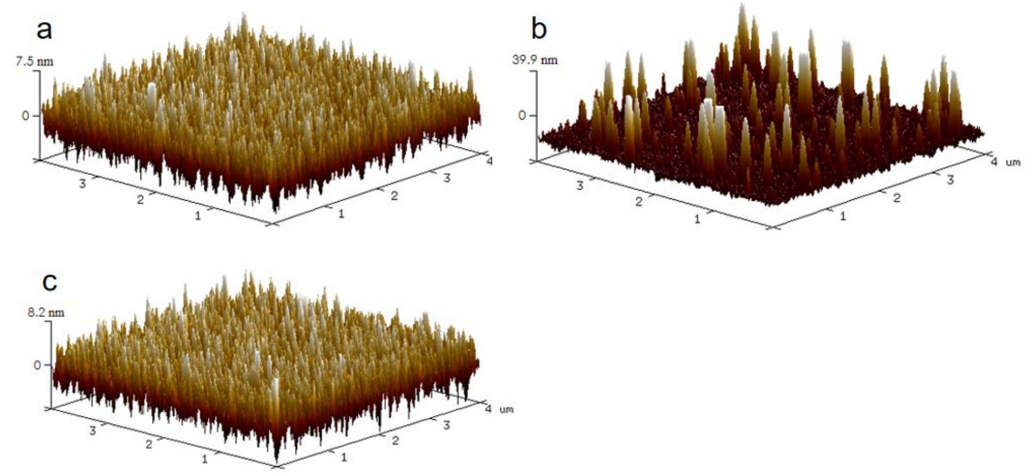

Figure S6. AFM images of MIM capacitors deposited with 12.5 nm Al_2O_3, TiO_2, HfO_2 dielectric material, respectively. (a) ALD deposition of Al_2O_3 dielectric material, (b) ALD deposition of TiO_2 dielectric material, (c) ALD deposition of HfO_2 dielectric material.

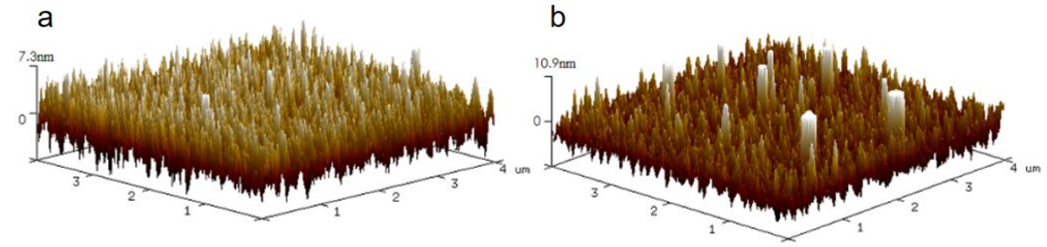

Figure S7. AFM images of MIM capacitors deposited with 50 nm Al_2O_3, TiO_2 dielectric material, respectively. (a) ALD deposition of Al_2O_3 dielectric material, (b) ALD deposition of TiO_2 dielectric material.
